# 5-Hydroxymethylfurfural Formation in Bread as a Function of Heat Treatment Intensity: Correlations with Browning Indices

**DOI:** 10.3390/foods10020417

**Published:** 2021-02-13

**Authors:** Gabriella Giovanelli, Carola Cappa

**Affiliations:** Dipartimento di Scienze per gli Alimenti, la Nutrizione e l’Ambiente, Università degli Studi di Milano, Via G. Celoria, 2-20133 Milano, Italy; gabriella.giovanelli@unimi.it

**Keywords:** bread, Maillard reaction products, HMF, browning, colour, regression models

## Abstract

5-hydroxymethylfurfural (HMF) is formed during bread baking as a Maillard reaction product (MRP); it can exert toxicity and it is regarded as a potential health risk because of its high consumption levels in western diets. The aim of this study was to evaluate HMF formation in bread as a function of heat treatment intensity (HTI) and to investigate correlations between HMF and easily detectable browning indices. White breads were baked at 200 °C and 225 °C for different baking times for a total of 24 baking trials. Browning development was evaluated by reflectance colorimetric and computer vision colour analysis; MRP were quantified spectrophotometrically at 280, 360 and 420 nm and HMF was determined by HPLC. HMF concentrations varied from 4 to 300 mg/kg dw. Colour indices (100–L*) and Intensity mean resulted significantly correlated between each other (*r* = −0.961) and with MRP (*r* ≥ 0.819). The effects of the different HTI were visualized by principal component analysis and the data were used to evaluate the best fitting regression models between HMF concentration and other browning indices, obtaining models with high correlation coefficients (*r* > 0.90).

## 1. Introduction

Bread is one of the most consumed foods worldwide and it can be produced using different ingredients and processing techniques. The baking phase is crucial, contributing to dough leavening and to the development of the desired colour, flavour, and texture characteristics. Crust appearance and flavour are the first quality requisites of bread evaluated by the consumer and which determine his preferences. During thermal processing, a variety of reactions take place, including protein denaturation and starch gelatinization in the crumb and the formation of the crust; this latter is due to dehydration and to the occurrence of reactions that ultimately lead to the development of the typical colour and aroma. Colour development during the baking process is generally referred to as browning, and is a consequence of physical phenomena, i.e., heat and mass transfer, that take place simultaneously during bread baking. The phenomenology and modelization of this process have been the object of various studies and were exhaustively reviewed by Purlis [1]. During bread baking, water evaporates from the product surface generating an evaporation front that progressively moves towards the core. As a consequence, in the outer layer water content decreases and temperature rises above 100 °C, asymptotically tending to the oven temperature. These changes lead to the formation of the crust, a region characterised by low moisture content (5–10%) and typical structural characteristics that avoid further dehydration of the crumb, where moisture does not substantially change and temperature remains around 100 °C [1].

The reactions involved in browning are the Maillard reaction and caramelization, whose rate and extent are affected by the formulation (presence and amount of reactants, pH, water availability) and the baking conditions (temperature, time, relative humidity, heating mode). In the Maillard reaction, reducing sugars react with amino compounds (amino acids, proteins and other nitrogen compounds) to form initial condensation products that undergo further transformations. The reaction develops in three stages: in the initial stage colourless sugar-amine condensation compounds are formed, such as the Amadori and Heyns compounds; in the intermediate stage colourless or yellow compounds with high UV adsorption are formed; in the final stage these compounds undergo further transformations up to the formation of high-molecular weight coloured endproducts called melanoidins [2]. Products formed in the intermediate and final stages through Strecker degradation are responsible for flavour and aroma development [3] and comprise typical heat-induced products such as acrylamide (AA) and 5-hydroxymethylfurfural (HMF). Caramelization does not involve nitrogen compounds; it is a consequence of dehydration of carbohydrates, in particular sucrose and reducing sugars, due to high temperature and favoured by low water activity and acidic pH [4]. These reactions lead to the formation of various compounds, among which HMF and furfural; the development of the reaction results in the formation of polymeric compounds with the typical brown colour [4]. 

Maillard reaction products (MRP) as well as caramelization products can exert both beneficial and detrimental effects from a nutritional point of view. Neo-formed heat-induced substances can play a positive role, showing antioxidant, antimicrobial and anti-inflammatory effects. On the other hand, browning reactions are related to a loss of nutritional value (loss of lysine, thermal degradation of other amino acids and reduced bioavailability of proteins) and, more worryingly, to the formation of mutagenic, carcinogenic and cytotoxic compounds [2,5,6,7].

Most concern and literature data are focused on AA, a low molecular weight compound derived from the Maillard reaction that has been detected in a wide range of foods, comprising cereal-based products such as bread, biscuits and breakfast cereals. AA is mainly formed from sugars and asparagine during severe heat treatment (i.e., roasting, baking, frying) and has been added to the list of neo-formed food contaminants since 2002; the European Union Commission set a series of regulatory measures to control and mitigate AA concentration in foodstuffs. Occurrence, toxicity and regulatory requisites concerning AA in food products have been recently reviewed [8]. 

HMF is a low molecular weight molecule formed in the second stage of the Maillard reaction [2] as well as by caramelization [4]. HMF concentration in heat-treated foods is related to the severity of the heat treatment and to other factors, principally type of sugar in the matrix, pH, water activity, presence of certain amino acids [1,5,6,9,10]. HMF can also form at relatively low temperatures in acidic foods, therefore it is evaluated as an index of time-temperature processing and storage conditions in products such as jams, fruit juices, tomato preserves [6,11]. HMF has been shown to exert toxicological activity in animals and cultured mammalian cells; literature data about detrimental health effects and dietary exposure to HMF have been recently reviewed [5,11]. Though toxicity for humans is still under investigation, HMF is regarded as a of neo-formed food contaminants since it can be converted in vivo into the genotoxic compound 5-sulfoxymethylfurfural [6]. One of the reasons to consider HMF as a potential health risk is its high concentration in a number of foods typically consumed in western diets, especially coffee, brown sugar and caramels, baked products and breakfast cereals [5,11,12]. Researchers conclude that since HMF levels of assumption are much higher than those of other food contaminants and may reach the biologically effective concentration, more data about distribution and concentration of HMF in foods is needed to control dietary exposure and related health risks. 

Established analytical techniques for the quantification of AA and HMF in foods are time-consuming and require proper analytical instrumentation and qualified operators, therefore they are impractical for routine process and quality control. Both AA and HMF concentrations in baked products are directly related to the browning degree [1,6,9]; evaluation and assessment of correlations between easily detectable heat-induced indices and neo-formed food contaminants could effectively help to control their level in baked foods. The aim of the current study was to evaluate HMF formation during bread baking as a function of the heat treatment intensity and to investigate correlations between HMF and other easily detectable heat-induced indices, such as colour and spectrophotometric parameters. The correlation models built on specific and standardized products could be used to estimate HMF concentration in a simple, economical and rapid way.

## 2. Materials and Methods

### 2.1. Materials

Soft wheat flour (Type 00, Carrefour, MI, Italy), compressed baker’s yeast (Carrefour, MI, Italy) and oil (Farchioni, Giano dell’Umbria, Italy) were purchased at a local supermarket and stored at 4 °C until use; yeast was used within the first two days after purchase. 

5-hydroxymethyl-2-furfural was from Sigma-Aldrich Italy; Carrez clarification kit was from Merck KGa, Darmstadt, Germany; all other reagents were analytical or HPLC grade.

### 2.2. Bread-Making and Baking Protocol 

A simple bread recipe was chosen in order to focus the attention on the effect of baking conditions instead of the raw material used. Preliminary trials were carried out to define baking conditions such as to produce underbaked and overbaked edible bread. During these trials, a temperature control system (WT210, Yokogawa Italia, Novate Milanese, Italy) equipped with three K thermocouples (Tersid, Milano, Italy) was used to measure the temperature inside the oven. The temperature fluctuation recorded was ±5 °C with respect to the set temperature. 

Each sample was identified by a code (Table 1) indicating the baking temperature, referred to as LT (lower temperature, 200 °C) and HT (higher temperature, 225 °C), and the baking time (minutes). Each baking trial was repeated twice in different days and the order of experiments was randomized to avoid the systematic errors and to minimize the effects of external factors.

The dough kneading was performed using a Hobart N-50 mixer, equipped with a spiral dough hook (Hobart Corporation, Troy, OH, USA). Wheat flour (400 g) was pre-mixed for 1 min at 136 rpm, then NaCl (7 g dissolved in an aliquot of water), oil (5 mL), yeast (20 g, suspended in an aliquot of water preheated at 25 °C), and the remaining water (out of 225 g), were added in the order above reported within the first 2 min of mixing, and then kneaded for 3 min more, at room temperature (approximately 20–25 °C). At the end of this period, the mixing was interrupted, the dough manually scraped from the surface of the bowl and mixed again for 5 min. The whole process lasted 11 min (1 min of pre-mixing, 10 min of kneading). After kneading the dough was collected, divided into 5 aliquots (60 g each), carefully hand modelled and positioned into rectangular baking moulds (10 × 6 × 4.5 cm). Few drops of vegetable oil were spread on the bottom of the baking moulds with a paper towel to facilitate bread unmoulding after baking. The doughs were leavened at 35 °C for 35 min using the leavening function of a multifunction oven (Whirlpool mod. AMW698/IXL, EMEA S.p.A, Biandronno, VA, Italy); a pan containing hot water (approximately 250 g of water preheated at 100 °C) was put on the bottom of the oven to increase the chamber humidity. The five breads were then baked in the oven (Whirlpool mod. G2551MF816A, EMEA S.p.A, Biandronno, Italy) according to the conditions reported in Table 1; the oven was preheated at the set temperature for at least 15 min, static function was used and moulds were positioned on the plate at medium level. At the end of baking, the samples were cooled at room temperature for 20 min before unmoulding, and then 5 min more. Breads were then evaluated for physical and chemical characteristics. 

### 2.3. Bread Characterization and Sample Homogenization

All bread samples (*n* = 5) were characterized for weight (g), maximum height (mm) measured by a calliper, and percentage baking loss calculated as (dough weight − bread weight) × 100/dough weight. Three of the five loaves were randomly selected and analysed for colour (Section 2.4). 

After colour evaluation, three bread halves (one for each bread) were pooled together and finely homogenized with a Braun mixer (Multiquick Zk100 4250, Braun GmbH, Kronberg im Taunus, Germany) for 30 s at room temperature. Homogenized samples were packaged in sealed containers and stored at 4 °C until MRP and HMF analysis (within 24 h).

Moisture content was determined gravimetrically by drying the homogenized sample at 105 °C to constant weight [13]. The analysis was carried out in triplicate (*n* = 3).

### 2.4. Colour Evaluation

Colour was measured on three of the five loaves (randomly chosen) from each baking trial.

**Figure 1:**
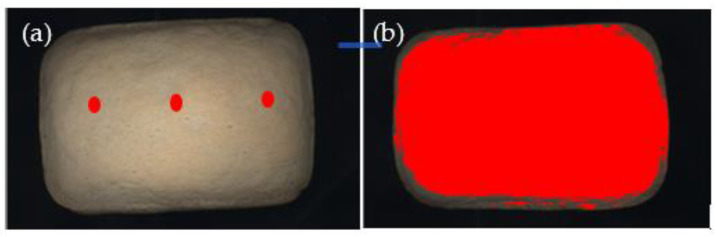
Bread surface area evaluation by colorimeter (**a**) and computer vision image analysis (**b**)**.**

CIELab colour was measured using a colorimeter (Chroma Meter II Reflectance, Minolta, Osaka, Japan), applying the head of the colorimeter (tip of the head was 8 mm in diameter) directly to the sample surface. The illumination system consisted of high-power pulsed xenon arc lamp, using C illuminant. Before measurements, the colorimeter was calibrated using the standard-white reflector plate (Y = 87.7; x = 0.308; y = 0.315). Results are given in the CIELab space, as L* (lightness, from 0 = black to 100 = white), a* (>0 = redness, <0 = greenness) and b* (>0 = yellowness, <0 = blueness); the browning index (BI, 100-L*) was also calculated [14]. For each baking trial, crust colour was evaluated measuring the top of three loaves in three points (right, middle and left; Figure 1a). After image-analysis, each bread was vertically cut in half and crumb colour was measured in the centre of the two halves. Results are the average of nine (3 loaves × 3 points, *n* = 9) and six (3 loaves × 2 halves, *n* = 6) measurements for crust and crumb, respectively. 

RGB colour was evaluated by computer vision image analysis on the images of the top crust and of the crumb of the three breads, acquired by Epson Perfection scanner (V850 pro, Seiko Epson Corporation, Suwa, Japan). In order to avoid light interferences samples were covered with a black box during the image acquisition. The images were acquired at 600 dpi resolution and 24 bit colour depth, acquiring the entire surface of the object. Before image acquisition, the scanner was calibrated with SilverFast 8 IT8 Colour Targets system (LaserSoft Imaging Inc., Kiel, Germany). Since the RGB model colour was selected, each pixel of the image was defined by the coordinates on three axes: R (red, from 0 to 255), G (green, from 0 to 255), and B (blue, from 0 to 255). Images were saved in TIFF format and processed using a dedicated software (Image Pro-Plus v. 4.5.1.29/XP; Media Cybernetics Inc., MD, USA). During image pre-processing a spatial calibration (600 dpi = 23.64 pixel/mm) was performed, to obtain images with the same size of the original samples; then images were segmented in order to select the object to analyse (i.e., bread) and exclude background and shadows at the borders of the object. Image segmentation was performed by setting the minimum area (100 mm^2^) of the object and the threshold value of 60 for B coordinate; no threshold values were set for R and G coordinates. The segmentation process allowed to identify the sample belonging to the foreground and to reject all the other objects (pixels) belonging to the background (Figure 1b). No image filters were applied. The following indices were considered: density red (R), density green (G) and density blue (B), and Intensity mean (IM), that represents the average of RGB channels in the raw data ((R + G + B)/3). Results are given as the average of the three breads’ measurements (*n* = 3).

### 2.5. Determination of Maillard Reaction Products

MRP were determined according to Delgado-Andrade et al. [15]. Briefly, 1 g homogenized sample (Section 2.3) was weighted in 40 mL centrifuge tubes, 20 mL distilled water was added and the tubes were vortexed for 15 s (1600 rpm). The tubes were then sonicated for 10 min, then vortexed again for 15 s. Samples were finally centrifuged at 14,000 g for 10 min at 4 °C and supernatants were sequentially filtered through Whatman N. 4 and 0.45 μm filters. The clear solutions were suitably diluted with distilled water and Absorbance at 280, 360 and 420 nm was measured using 1 cm optical path quartz cuvettes (V-650 spectrophotometer, Jasco Europe). Each extraction was carried out in triplicate and each extract was singly analysed (*n* = 3). Due to the unavailability of standard compounds to calibrate the method, data are expressed as absorbance units referred to 1 g of dry weight sample (AU/g dw), in order to consider different moisture contents. 

### 2.6. Determination of 5-hydroxymethylfurfural

Extraction and analysis of HMF were performed by the method described by Delgado-Andrade et al. [15] with some modifications. 1 g of homogenized sample (Section 2.3) was weighted in a 40 mL centrifuge tube ad 19 mL distilled water was added. Samples were vortexed for 1 min (1600 rpm), 0.5 mL Carrez I solution was added and the mixture was vortexed for 10 seconds, 0.5 mL Carrez II solution was added and the mixture was vortexed for 10 seconds. The tubes were then centrifuged at 14,000 g at 4 °C for 10 min. Supernatants were filtered (Whatman N. 4), then microfiltered (0.45 μm acetate filters) and samples injected in the HPLC system (Pump L-7100, UV detector L-7400, Merck, Darmstadt, Germany). A Spherisorb ODS-2 column (5 μm, 4.6 mm × 250 mm) was used, the elution phase was water:acetonitrile (95:5) 1 mL/min and elution temperature was 32 °C. Chromatograms were recorded at 280 nm and HMF was identified and quantified through a calibration curve built with the pure standard compound (Sigma-Aldrich Italia). Each sample was extracted in triplicate and each extract was singly analysed (*n* = 3). Data are expressed as mg/kg dw. 

### 2.7. Statistical Analysis

Analytical results were processed by one-way analysis of variance (ANOVA), followed by the Least Significant Difference (Fisher’s LSD) test to highlight significant differences (*p* < 0.05) among samples. The relationship between the measured indices was assessed by Pearson linear correlation. In order to estimate HMF as a function of browning indices, different regression models were explored. All data were processed by Statgraphics Centurion (v. 18, Statistical Graphics Corp., Herndon, VA, USA). 

Data related to bread browning, HMF and MRP were explored by principal component analysis (PCA) performed by The Unscrambler software package (v. 9.7, Camo Software AS, Oslo, Norway).

## 3. Results and Discussion

### 3.1. Bread Characterization

Table 2 shows bread height, percentage baking loss and moisture content of the differently baked breads. Data evidence that increasing time-temperature conditions produce higher weight losses and corresponding lower moisture content in the baked products. Average weight loss in bread baking is around 25% and for the Italian low bread moisture cannot exceed 29% for loaves weighting up to 70 g [16]. Therefore, although no limits are set regarding minimum moisture content and colour development in bread, we can consider that typical baking conditions in our experiments were exceeded for times longer than 37.5 min at 200 °C (moisture content 21.1–23.2 g/100g and percentage baking loss 26.3–26.6%) and 30 min at 225 °C (moisture content 21.9–23.0 g/100 g and percentage baking loss 27.0–27.6). Final bread developing, as determined by bread height, was not influenced by the baking conditions; the results of statistical analysis (Table 2) show minor differences between samples, that do not depend on the baking intensity. 

### 3.2. Colour Development

**Figure 2:**
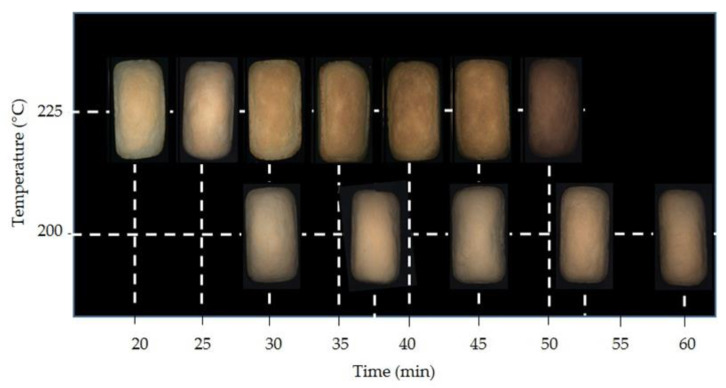
Images of bread samples baked at 200 °C and 225 °C for different baking times.

**Figure 3:**
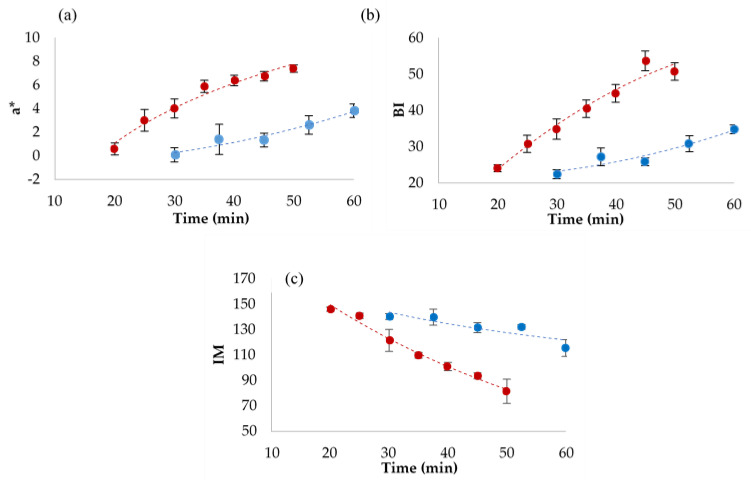
Redness (**a**), browning index (**b**) and intensity mean (**c**) of bread crust as a function of baking time at 200 °C (●) and 225 °C (●). Average of replicate trials, bars represent standard deviation.

The development of browning is a typical characteristic of baked products. Two techniques were used to evaluate colour: colorimetric analysis by reflectance colorimeter, which provides point-like information in the L*a*b* colour space, and image analysis by computer vision, which provides results obtained by processing a very large number of data, in this case the whole surface of the breads (Figure 2); colour data obtained by image analysis are given as RGB colour indices. The colour parameters obtained for all the baking trials are reported in Table S1. Bread crust colour varied from light to darker according to baking time and temperature, as can be appreciated by the photographs in Figure 2. As evidenced by the data in Table S1, the more representative indices of crust colour changes were a* and L* or the browning index (BI, 100 – L*) in the CIELab colour space; in the RGB space, all coordinates showed a decrease related to the baking time and all the information is synthetized in the intensity mean (IM, (R+G+B)/3) value. The evolution of a*, BI, and IM are presented in Figure 3, where the results obtained in the two replications were averaged to show the overall colour trend; bars represent standard deviation of data. As expected, colour changes were faster and larger in the breads baked at 225 °C.

The a* colour index (Figure 3a) increased with increasing baking time at both temperatures, rising from 0.15 to 3.85 at 200 °C and from 0.55 to 7.40 at 225 °C, indicating a colour change towards higher redness, typical of baked products. The browning index (Figure 3b) had a similar behaviour, due to the decrease of L* at increasing baking times. L* corresponds to luminosity and ranges from 100 (white) to 0 (black), and its decrease means a general darkening of the product. As expected, colour changes were larger at 225 °C (maximum BI = 55,9, Table S1). Browning development was much slower at 200 °C (maximum BI = 35.5, Table S1). The evolution and extent of browning observed in our experiment are in accordance with previously reported data [1,15]. Figure 3c shows colour changes in terms of IM; in this case, high values correspond to light colour and low values correspond to dark colour. This index is representative of the whole bread surface colour being obtained by image analysis. As expected, IM decrease was faster and larger at 225 °C (minimum IM = 72.8 ± 0.9) than at 200 °C (minimum IM = 109.5 ± 2.1), reproducing an evolution opposite to BI. Accordingly, BI and IM were negatively correlated (*r* = −0.961; *p* < 0.0001). The good correlation allows us to conclude that both colorimetric reflectance analysis and computer vision colour analysis are suitable techniques to measure bread colour and the development of browning. However, in the case of non-homogenous surface colour, the point-like colour measurement is less reliable, as previously reported for round cookies [17].

Data in Table S1 show that colour of the crumb was not influenced by the baking conditions, even at the highest time-temperature profile. Browning development is highly related to the oven temperature and water activity of the product, and these parameters do not significantly change in the inner part of bread (crumb), whose temperature is steadily just below 100 °C and water activity does not substantially change, unless a toasting process is achieved [1]. The physical-chemical phenomena related to browning development in bakery products were widely discussed by Purlis [1] and our data match with the described phenomena and with data in the literature [14,18,19,20]. In particular, experimental data obtained from baking tests carried out on bread and other baked products indicate that the initiation of browning requires minimum temperature of 105–120 °C and water activity values below 0.4–0.7. The crust of white bread baked with oven temperature from 180 °C to 220 °C for times up to 30 min, reached final temperatures ranging from 140 °C to 180 °C and water activity values (obtained by numerical modelization) in the range of 0.05–0.2 [1]. These conditions favour the desired browning reactions as well as the formation of neo-formed food contaminants.

### 3.3. Maillard Reaction Products (MRP)

The formation of MRP was followed by measuring absorbance of the aqueous extracts of the homogenized breads (crust and crumb) at 280, 360 and 420 nm [15]; therefore, the amounts detected are representative of the whole bread. These wavelengths respectively correspond to maximum absorbance of non-coloured low molecular weight compounds generated in the initial steps of the Maillard reaction, more advanced products with intermediate molecular weight, and coloured high molecular weight compounds, which are generally referred to as melanoidins.

**Figure 4:**
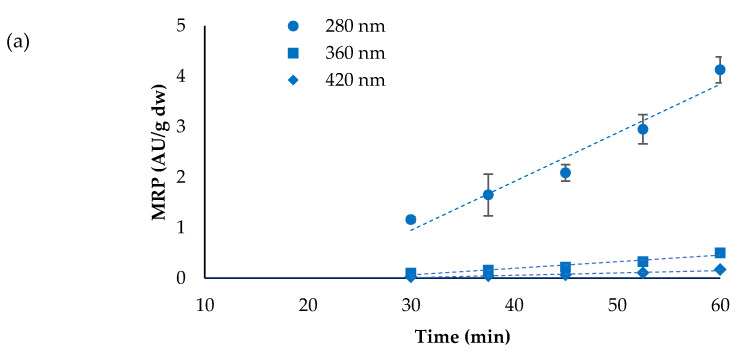

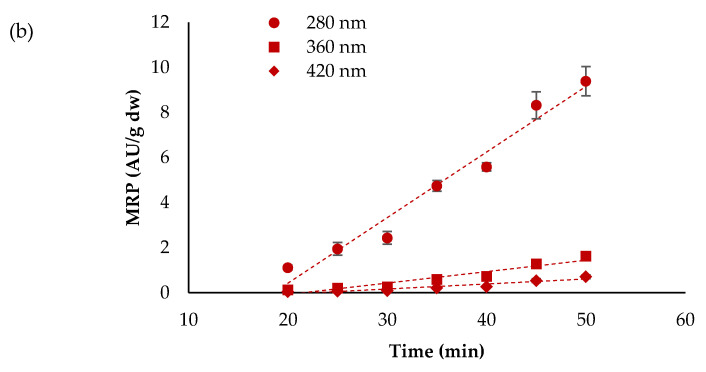
Evolution of Maillard reaction products (MRP) determined at 280, 360 and 420 nm in bread baked at 200 °C (**a**) and 225 °C (**b**). Average of replicate trials, bars represent standard deviation.

Data in Table S2 and graphs in Figure 4 show the evolution of MRP related to early, intermediate and advanced stages of the reaction. The concentration of MRP increased almost linearly at both baking temperatures, with similar trends at the three wavelengths. Increase in MRP was clearly faster at 225 °C than at 200 °C. Absorbance at 280 nm, corresponding to low molecular weight, non-coloured MRP, reached an average value of 9.38 AU/g dw in the bread baked at 225 °C for 50 min, which was more than twice the value observed in the sample baked at 220 °C for 60 min (4.12 AU/g dw). Even higher ratios were found for AU values at 360 and 420 nm (intermediate and advanced coloured MRP), which were approximately 3 times higher (average values 1.61 *vs*. 0.50 AU/g dw) and 4 times higher (average values 0,70 *vs*. 0.17 AU/g dw) in the bread baked at 225 °C for 50 min than in the sample baked at 220 °C for 60 min. These data demonstrate the primary effect of temperature on the Maillard reaction kinetics, especially for the advanced stages, which are more accelerated by a temperature increase. 

MRP evaluated at 280, 360 and 420 nm were positively correlated with the BI (linear correlation, *r* = 0.951, *r* = 0.819, *r* = 0.856, respectively; *p* < 0.0001), and with the IM (linear correlation, *r* = −0.951, *r* = −0.824, *r* = −0.862, respectively; *P* < 0.0001) confirming the relationship between colour browning and MRP concentration. Contrarily to what is reported by Helou et al. [18], we can also conclude that the spectrophotometric method used in this work is appropriate for the determination of MRP concentrations in bread samples, as results appear consistent with colour development.

### 3.4. HMF Formation

**Figure 5:**
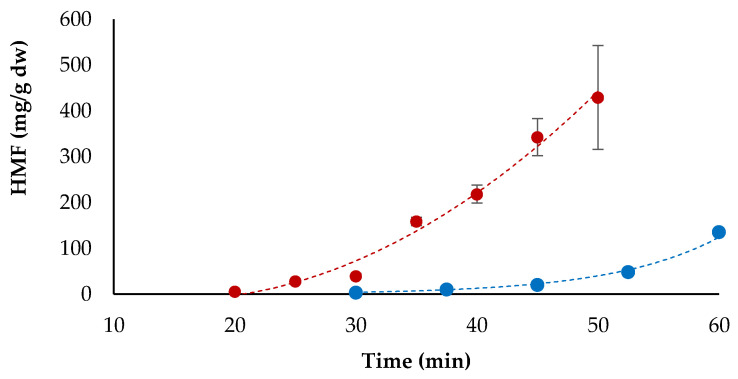
Evolution of 5-hydroxymethylfurfural (HMF) in bread baked at 200 °C (●) and 225 °C (●). Average of replicate trials, bars represent standard deviation.

HMF was analysed in the homogenised samples (crust and crumb); therefore, the amounts detected are representative of the whole bread. Although HMF is mainly formed in the crust and only traces can be detected in the crumb [14,18], we decided to analyse the combination of crust and crumb because of the difficulty to separate the two fractions in a standardised and reproducible manner, and in order to provide information on HMF concentration in the product as it is consumed. The amounts of HMF in the experimental breads are reported in Table S2, while Figure 5 shows HMF evolution at 220 °C and 225 °C considering averaged data. HMF production was highly influenced by the baking temperature and showed a parabolic evolution trend (Figure 5). Kinetics of HMF formation were similar to those observed for the MRP (Figure 4), as it was expected. Concentrations in the experimental breads varied widely, from approximately 4–6 mg/kg dw at the shortest baking times (30 min at 200 °C and 20 min at 225 °C) to more than 300 mg/kg dw after 45 and 60 min at 225 °C; the highest value in bread baked at 200 °C was 140.8 mg/kg dw (Table S2). The concentration values detected in our samples are in the range of values reported in the literature for breads and baked products: HMF values ranged from 3.4 to 68.8 mg/kg dw in Spanish white bread [14] and from 2.7 to 132.9 mg/kg in Turkish breads, with the highest values in breads baked in stone oven [21]; HMF values ranging from 6 to 210 mg/kg were reviewed in various cereal-based products [11]. 

### 3.5. Principal Component Analysis and Regression Models

**Figure 6:**
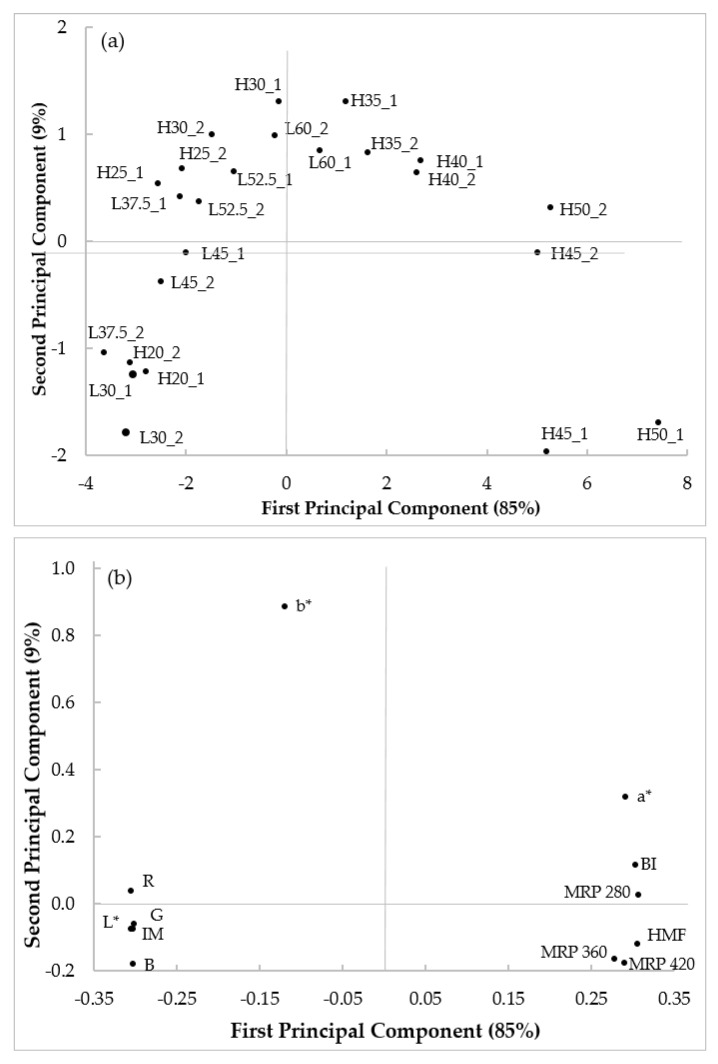
Principal component analysis score plot (**a**) and loadings plot (**b**) of bread browning, HMF and Maillard reaction product (MRP) data collected on bread samples baked at 200 °C and 225 °C for different baking times. For sample identification see (Table 1); a*, redness; b*, yellowness; B, density blue; BI, browning index; G, density green; HMF, 5-hydroxymethylfurfural; IM, Intensity mean; L*, lightness; MRP 280, Maillard reaction products evaluated at 280 nm; MRP 360, Maillard reaction products evaluated at 360 nm; MRP 420, Maillard reaction products evaluated at 420 nm; R, density red.

Figure 6 shows the PCA score and loading plots obtained with colour indices, HMF and MRP data collected on bread samples baked at 200 °C and 225 °C for the different baking times. The PCA score plot (Figure 6a) indicates a clear evolution of samples along the first principal component (PC1, 85% of explained variance) and the second principal component (PC2, 9% of explained variance) according to the heat treatment intensity. The PCA loading plot (Figure 6b) indicates that all the variables investigated have high positive or negative loading values, thus accounting for sample distribution. In particular, most of the variables have high positive or negative values of PC1, with the exception of b*, which has a high weight on PC2. 

In general, samples baked at low temperature for short baking time (i.e., L30) have negative values of PC1 and PC2, and they are characterized by higher RGB colour coordinates and L* values. As the conditions become more severe (i.e., low temperature for longer time or high temperature for short time), samples move to the upper right part of the plot (higher b* values) and then they progressively move down to the right, where samples are described by high values of BI, HMF and MRP (evaluated at 280, 360 and 420 nm) and low values of IM. This behaviour confirms what already discussed in the previous sections: temperature affects bread browning more than time, but similar sample evolution on the PCA plane was observed at 200 and 225 °C. 

Furthermore, the PCA score plot can be used to summarize and visualize the effects of the different baking treatments: for instance, samples baked at 200 °C for 30 and 37.5 min (L30 and L37.5) are similar (i.e., closely located) to the sample baked at 225 °C for 20 min (H20); a baking time of 25 min at 225 °C resulted comparable to a treatment twice as long (52.2 min) at 200 °C; lastly, samples baked at lower temperature for the longest baking time (L60) showed heat-induced changes similar to samples baked at higher temperature for relatively short time (H30). 

The development of the Maillard reaction in food products depends on many factors and various kinetic models of bread browning are discussed in the literature [22,23]. Kinetic models were suggested to investigate the effects of type of sugars (glucose, fructose, sucrose and glucose + fructose) on acrylamide and HMF formation in biscuits [10] and to estimate the browning degree in bread using a non-isothermal approach, considering the influence of water activity and baking temperature [22]. 

**Figure 7:**
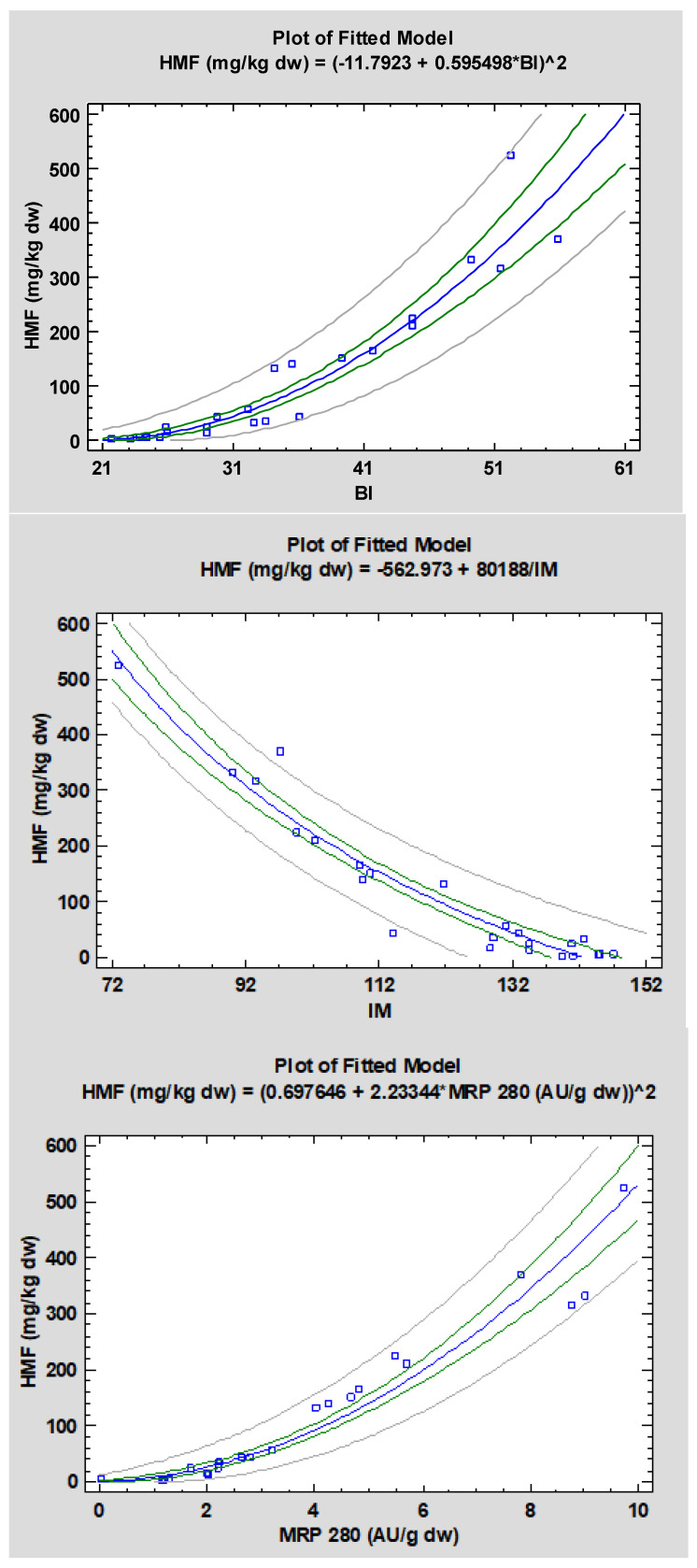

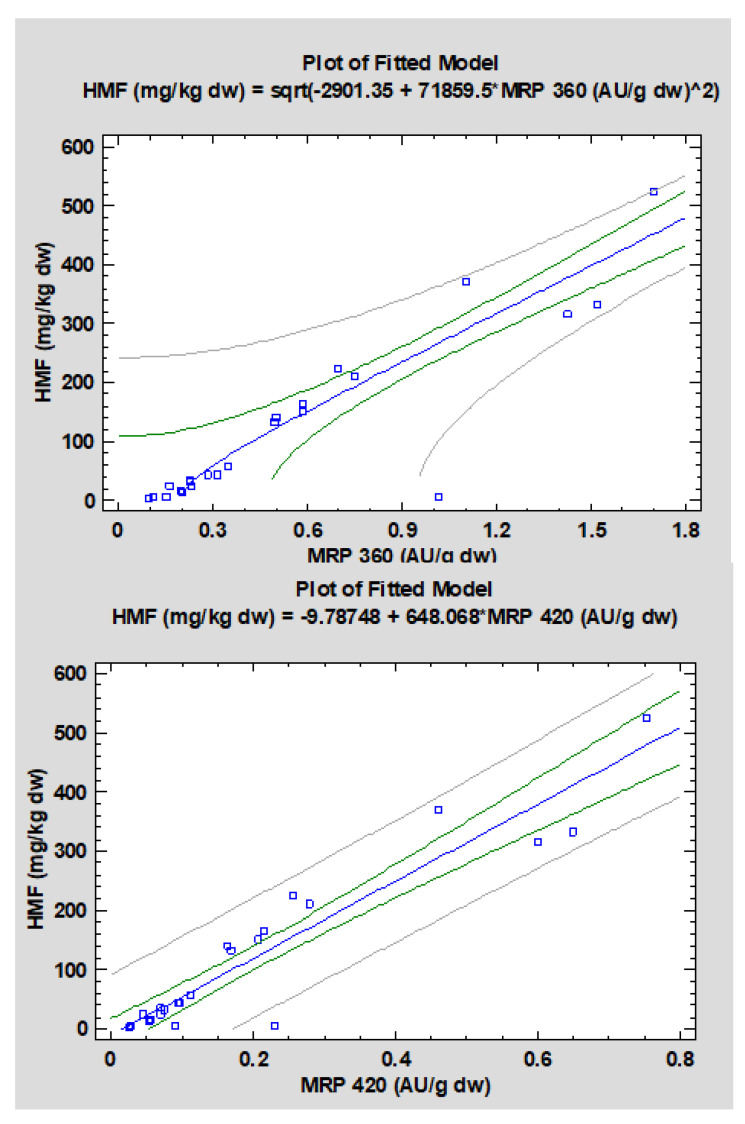
Best fitting regression models for the estimation of 5-hydroxymethylfurfural (HMF) in bread samples as a function of colour and spectrophotometric indices. BI, browning index; IM, Intensity mean; MRP 280, Maillard reaction products evaluated at 280 nm; MRP 360, Maillard reaction products evaluated at 360 nm; MRP 420, Maillard reaction products evaluated at 420 nm.

In the current study, in order to provide a tool to estimate HMF concentration, simple regression models between HMF and other easily detectable indices (i.e., colour and spectrophotometric parameters) were explored. All the data in pool were used to create the regression curves, as the objective was to obtain models to be used independently on the baking time and temperature. Linear correlations between HMF and BI were previously reported for common, special and snack breads [14,21]; though correlations were significant (*r* > 0.7, *p* < 0.05), they were based on very few data (3 and 5 bread samples for [14] and [21], respectively). Using the pooled data obtained in our study (*n* = 24), HMF was linearly correlated with BI (Equation Y = −335.531 + 13.0099X, *r* = 0.930), IM (Equation Y = 901.828 − 6.44202X, *r* = −0.940) and MRP (Equation Y = −68.0249 + 50.2243X, *r* = 0.969, and Equation Y = −25.162 + 268.004X, *r* = 0.898, for 280 and 360 nm, respectively), obtaining highly significant correlations. Non-linear models were also explored and the models yielding the highest R-Squared value were selected. The results of fitting are listed in (Table 3) and the corresponding curves are shown in (Figure 7). P-values were always lower than 0.0001 and the high correlation coefficients (*r* > 0.90) indicate a strong relationship between the variables. Furthermore, according to *R*-Squared values, the models explain from a minimum of 81.2% to a maximum of 95.6% of the variability in HMF. Figure 7 shows the best fitting regression models (blue line) with the indication of the prediction limit (grey lines) and the confidence limit (green lines). In general, for HMF values <100 mg/kg dw, a linear relation can be observed, but as the browning reaction proceeds, linear models are only adequate to estimate HMF as a function of coloured high molecular weight compounds (MRP evaluated at 420 nm), whereas non-linear regression models better fit the other variables. These correlations apply to the specific product used in this study; with a similar approach, correlations can be built and used for the assessment of quality parameters in other heat-treated products. Other researchers have drawn similar conclusions with regard to AA estimation in biscuits and potato chips [24].

## 4. Conclusions

The results obtained in this work confirm the direct relationship between the intensity of the heat treatment and the development of the Maillard reaction in bread baking. The evolution of the colour indices and the analytical parameters considered in the study (MRP and HMF) was clearly related to the baking temperature and time; data show that temperature affected bread browning more than time, but similar trends were observed at 200 and 225 °C. The colour indices determined by reflectance colorimeter (CIELab colour space) and by computer vision image analysis (RGB colour space) were highly correlated between each other, indicating that in the case of sufficiently even surfaces (such as plain white bread) both methods can be effectively used to acquire colour data. The regression models that correlate HMF concentration with colour indices (BI and IM) and MRP content (determined by absorbance at 280, 360 and 420 nm) of bread samples showed high correlation coefficients (*r* > 0,900, *p* < 0.0001). A limit of the present study is that such correlations are necessarily related to a specific product, characterized by specific formulation, size and baking mode, as these parameters have an influence on browning and HMF formation. In our study, a plain white bread was used and two baking temperatures were investigated; when considering specific and standardized baked products, which is the case of many industrial and semi-industrial products, regression models similar to those obtained in this study could be built and represent an interesting tool to estimate HMF concentration in a simple, rapid and economic way.

## Figures and Tables

**Table 1:** Bread samples identification and baking conditions.

Sample Code	Baking Temperature (°C)	Baking Time (min)
L30	200	30
L37.5	200	37.5
L45	200	45
L52.5	200	52.5
L60	200	60
H20	225	20
H25	225	25
H30	225	30
H35	225	35
H40	225	40
H45	225	45
H50	225	50

**Table 2:** Height, baking weight loss and moisture content of bread samples (average value ± standard deviation for each replicated trial).

Sample Code *	Maximum Height (mm)	Baking Weight Loss (%)	Moisture Content (g/100 g)
L30_1	43 ± 2 ^fgh^	23.1 ± 0.4 ^b^	25.9 ± 0.1 ^kl^
L30_2	41 ± 1 ^cde^	23.4 ± 0.8 ^bc^	25.4 ± 0.5 ^k^
L37.5_1	43 ± 2 ^fgh^	26.3 ± 0.7 ^d^	21.1 ± 0.3 ^h^
L37.5_2	42 ± 1 ^ef^	26.6 ± 0.5 ^de^	23.2 ± 0.4 ^j^
L45_1	38 ± 1 ^ab^	30.0 ± 0.2 ^gh^	20.0 ± 0.2 ^g^
L45_2	42 ± 1 ^ef^	31.9 ± 0.6 ^i^	18.3 ± 0.2 ^f^
L52.5_1	45 ± 3 ^hi^	32.9 ± 0.6 ^j^	16.8 ± 0.1 ^e^
L52.5_2	38 ± 1 ^ab^	34.2 ± 2.4 ^k^	16.6 ± 0.2 ^e^
L60_1	41 ± 1 ^def^	35.6 ± 1.0 ^lm^	12.5 ± 0.1 ^b^
L60_2	45 ± 1 ^ghi^	36.0 ± 0.7 ^m^	11.6 ± 0.1 ^a^
H20_1	40 ± 1 ^bcd^	19.8 ± 0.5 ^a^	28.6 ± 0.4 ^m^
H20_2	41 ± 1 ^cde^	19.8 ± 0.5 ^a^	28.5 ± 0.4 ^m^
H25_1	39 ± 2 ^bc^	23.2 ± 0.5 ^b^	26.2 ± 0.5 ^l^
H25_2	43 ± 1 ^fg^	24.0 ± 0.3 ^c^	25.7 ± 0.7 ^kl^
H30_1	40 ±1 ^bcd^	27.0 ± 0.6 ^e^	23.0 ± 0.5 ^j^
H30_2	40 ± 2 ^cd^	27.6 ± 0.3 ^f^	21.9 ± 0.6 ^i^
H35_1	40 ± 1 ^bcd^	29.4 ± 0.5 ^g^	18.5 ± 0.4 ^f^
H35_2	45 ± 1^i^	30.5 ± 0.6 ^h^	19.8 ± 0.3 ^g^
H40_1	39 ± 2^bc^	31.8 ± 0.2 ^i^	16.5 ± 0.1 ^e^
H40_2	41 ± 2 ^cde^	32.7 ± 0.2 ^j^	15.4 ± 0.3 ^d^
H45_1	48 ± 1 ^j^	35.0 ± 0.4 ^l^	14.2 ± 0.4 ^c^
H45_2	40 ± 2 ^cde^	35.0 ± 0.8 ^l^	14.1 ± 0.1 ^c^
H50_1	39 ± 1 ^bc^	37.7 ± 0.8 ^m^	11.3 ± 0.2 ^a^
H50_2	37 ± 2 ^a^	38.0 ± 0.3 ^n^	11.5 ± 0.1 ^a^

* For sample identification see Table 1; replicated trials are identified by _1 and _2; values followed by different letters in each column are significantly different (*p* < 0.05).

**Table 3:** Best fitting correlation models between 5-hydroxymethylfurfural (HMF) and colorimetric and spectrophotometric indices (pooled data).

Simple Regression * (Y; X)	Best Fitting Model	*r*	*p*-Values	Equation
HMF; BI	Square root-Y model	0.966	0.0000	Y = (−11.7923 + 0.595498X)^2^
HMF; IM	Reciprocal-X model	0.968	0.0000	Y = −562.973 + 80188/X
HMF; MRP 280	Square root-Y model	0.978	0.0000	Y = (0.697646 + 2.23344X)^2^
HMF; MRP 360	Double-squared	0.900	0.0000	Y = sqrt(−2901.35 + 71859.5X^2^)
HMF; MRP 420	Linear	0.945	0.0000	Y = −9.78748 + 648.068X

* BI, browning index; IM, Intensity mean; MRP 280, Maillard reaction products evaluated at 280 nm; MRP 360, Maillard reaction products evaluated at 360 nm; MRP 420, Maillard reaction products evaluated at 420 nm.

## Supplementary Materials

The following are available online at https://www.mdpi.com/2304-8158/10/2/417/s1, Table S1: Colour indices of bread samples (average value ± standard deviation for each replicated trial). Table S2: 5-hydroxymethylfurfural (HMF) and Maillard reaction products (MRP) content of bread samples (average value ± standard deviation for each replicated trial).
