# Supplementary material for: 5-Hydroxymethylfurfural Formation in Bread as a Function of Heat Treatment Intensity: Correlations with Browning Indices
Source: Foods. 2021 Feb 13;10(2):417. doi: 10.3390/foods10020417 (PMC7918450; doi:10.3390/foods10020417)
Supplement: Supplementary file 1 [file foods-10-00417-s001.pdf]

Table 1 supplementary material: Colour indices of bread samples (average value ± standard deviation for each replicated trial).
*For sample identification see Table 1; replicated trials are identified by _1 and _2; for crust and crumb, values followed

**Sample code***	**L***	**a***	**b***	**BI**	**R**	**G**	**B**	**IM**
Crust								
L30_1	76.9 ± 1.3^{mn}	0.2 ± 0.7^{ab}	24.9 ± 1.3^{bc}	23.1^{ab}	159.8 ± 2.1^{h}	142.1 ± 2.7^{jkl}	116.5 ± 3.0^{jk}	139.5 ± 2.1^{i}
L30_2	77.3 ± 0.6^{n}	0.1 ± 0.5^{a}	23.5 ± 1.5^{c}	22.7^{a}	159.9 ± 2.5^{h}	143.8 ± 2.2^{klm}	119.4 ± 2.1^{kl}	141.0 ± 2.5^{i}
L37.5_1	71.0 ± 2.1^{k}	2.3 ± 0.7^{e}	28.7 ± 0.7^{fghij}	29.0^{d}	160.9 ± 1.9^{hi}	135.8 ± 2.2^{h}	106.6 ± 2.3^{i}	134.4 ± 1.9^{h}
L37.5_2	74.6 ± 0.9^{l}	0.4 ± 0.9^{abc}	25.8 ± 1.6^{cd}	25.4^{c}	180.1 ± 6.0^{k}	163.9 ± 5.0^{o}	123.3 ± 5.9^{l}	145.1 ± 6.0^{j}
L45_1	74.1 ± 1.2^{l}	1.7 ± 0.4^{d}	27.3 ± 0.6^{defg}	25.9^{c}	151.2 ± 2.1^{g}	130.7 ± 2.4^{g}	104.1 ± 2.3^{hi}	128.7 ± 2.1^{g}
L45_2	74.2 ± 1.0^{l}	1.0 ± 0.6^{c}	27.2 ± 0.7^{cd}	25.8^{c}	164.5 ± 3.1^{hij}	145.2 ± 4.6^{lm}	106.5 ± 5.4^{i}	134.4 ± 3.1^{h}
L52.5_1	67.9 ± 2.4^{j}	3.0 ± 0.8^{f}	29.2 ± 0.4^{hij}	32.1^{e}	159.2 ± 2.3	131.7 ± 1.6^{gh}	101.4 ± 1.2^{gh}	130.8 ± 1.7^{gh}
L52.5_2	70.3 ± 1.3^{k}	2.2 ± 0.5^{e}	28.8 ± 0.7^{fghij}	29.7^{d}	159.6 ± 1.9^{h}	139.7 ± 1.1^{ijk}	100.6 ± 0.5^{gh}	133.0 ± 1.9^{h}
L60_1	64.5 ± 1.3^{gh}	3.9 ± 0.7^{gh}	29.0 ± 0.5^{ghij}	35.5^{gh}	135.1 ± 2.1^{de}	109.8 ± 1.6^{de}	83.8 ± 1.2^{ef}	109.5 ± 2.1^{d}
L60_2	65.9 ± 0.72^{h}	3.8 ± 0.4^{gh}	29.9 ± 0.7^{j}	34.1^{fg}	152.0 ± 2.9^{g}	127.8 ± 2.0^{g}	87.9 ± 1.3^{f}	121.6 ± 0.6^{f}
H20_1	75.7 ± 0.9^{lm}	0.6 ± 0.3^{bc}	26.3 ± 0.9^{cde}	24.3^{bc}	165.7 ± 2.0^{ij}	147.5 ± 0.7^{m}	121.5 ± 0.6^{l}	144.9 ± 0.8^{j}
H20_2	76.2 ± 1.0^{n}	0.5 ± 0.7^{bc}	26.0 ± 1.4^{cd}	23.8b	168.7 ± 2.1^{j}	153.5 ± 1.1^{n}	119.3 ± 0.9^{kl}	147.1 ± 1.1^{j}
H25_1	71.0 ± 1.4^{k}	2.4 ± 0.6^{e}	29.4 ± 0.8^{ij}	29.0^{d}	166.7 ± 2.2^{j}	142.8 ± 2.2^{jklm}	113.1 ± 2.1^{j}	140.8 ± 2.1^{i}
H25_2	67.4 ± 1.5^{ij}	3.6 ± 0.7^{g}	29.3 ± 0.5^{ij}	32.6^{ef}	164.3 ± 3.2^{ij}	148.8 ± 4.0^{mn}	110.6 ± 3.5^{j}	142.6 ± 2.8^{ij}
H30_1	63.9 ± 2.8^{g}	4.3 ± 0.9^{h}	29.6 ± 0.3^{j}	36.1^{h}	140.4 ± 3.9^{f}	114.5 ± 4.^{8f}	87.1 ± 4.2^{f}	114.0 ± 4.3^{e}
H30_2	66.5 ± 2.3^{ij}	3.7 ± 0.5^{gh}	29.6 ± 0.6^{j}	33.5^{ef}	161.6 ± 5.4^{hi}	138.6 ± 5.0^{ij}	98.8 ± 4.0^{g}	129.0 ± 2.1^{g}
H35_1	60.7 ± 1.6^{f}	5.6 ± 0.4^{i}	29.7 ± 0.5^{j}	39.3^{i}	139.5 ± 3.9^{ef}	109.8 ± 3.1^{de}	82.5 ± 2.3^{e}	110.6 ± 3.1^{de}
H35_2	58.3 ± 2.5^{e}	6.2 ± 0.4^{l}	27.9 ± 0.7^{efghi}	41.7^{j}	136.3 ± 1.1^{ef}	112.9 ± 0.7^{ef}	77.8 ± 0.6^{d}	109.0 ± 0.8^{d}
H40_1	55.3 ± 2.4^{d}	6.4 ± 0.5^{lm}	27.4 ± 1.2^{defgh}	44.7^{k}	126.3 ± 0.9^{c}	98.4 ± 0.7^{c}	74.2 ± 0.6^{cd}	99.6 ± 0.7^{c}
H40_2	55.3 ± 2.7^{d}	6.4 ± 0.4^{lm}	27.2 ± 1.5^{def}	44.7^{k}	130.1 ± 5.5^{cd}	105.8 ± 4.8^{d}	71.4 ± 3.4^{c}	102.4 ± 4.6^{c}
H45_1	44.1 ± 1.8^{a}	6.7 ± 0.3^{lm}	19.5 ± 1.5^{a}	55.9^{n}	114.9 ± 4.2^{b}	92.3 ± 2.6^{b}	68.4 ± 2.6^{bc}	97.2 ± 3.1^{bc}
H45_2	48.5 ± 1.2^{b}	6.8 ± 0.5^{mn}	26.0 ± 2.3^{def}	51.5^{m}	119.1 ± 2.9^{b}	96.6 ± 2.5^{bc}	65.1 ± 1.6^{b}	93.6 ± 2.3^{b}
H50_1	47.7 ± 1.5^{b}	7.5 ± 0.3^{o}	21.3 ± 7.4^{b}	52.3^{m}	95.3 ± 1.1^{a}	70.0 ± 1.1^{a}	53.2 ± 0.7^{a}	72.8 ± 0.9^{a}
H50_2	50.8 ± 2.1^{c}	7.3 ± 0.4^{no}	27.4 ± 1.9^{defg}	49.2^{l}	114.6 ± 1.6^{b}	93.2 ± 1.7^{b}	61.9 ± 1.8^{b}	90.0 ± 1.8^{b}
Crumb								
L30_1	76.6 ± 1.5^{j}	-2.8 ± 0.2^{abc}	15.3 ± 0.7^{ghi}	23.4^{a}	163.0 ± 4.4^{b}	151.1 ± 4.5^{c}	126.0 ± 4.3^{cd}	146.0 ± 4.4^{c}
L30_2	73.3 ± 2.1^{efg}	-2.9 ± 0.2^{abc}	15.2 ± 0.7^{fgh}	26.7^{def}	163.3 ± 3.1^{b}	152.0 ± 3.2^{c}	127.6 ± 2.9^{de}	147.6 ± 3.0^{c}
L37.5_1	72.9 ± 0.8d^{ef}	-3.4 ± 0.3^{abc}	13.8 ± 0.9^{cde}	27.1^{efg}	194.6 ± 1.9^{kl}	180.2 ± 2.0^{lm}	151.0 ± 2.1^{l}	175.3 ± 1.9^{l}
L37.5_2	74.0 ± 0.4f^{ghi}	-3.7 ± 0.3^{abc}	13.3 ± 0.6^{bc}	26.0b^{cde}	195.9 ± 2.5^{l}	183.8 ± .4^{m}	145.2 ± 2.3^{j}	175.0 ± 2.4^{l}
L45_1	75.5 ± 1.3^{ij}	-2.9 ± 0.2^{abc}	16.9 ± 0.4^{j}	24.6^{ab}	157.1 ± 1.2a	144.6 ± 1.1^{b}	119.7 ± 1.1^{b}	140.5 ± 1.0^{b}
L45_2	75.0 ± 0.5^{hij}	-4.0 ± 0.2^{a}	12.9 ± 0.5^{b}	25.0^{abc}	188.3 ± 2.2^{ghi}	176.3 ± 1.9^{jkl}	139.0 ± 2.2^{hi}	167.9 ± 2.1^{ghi}
L52.5_1	71.4 ± 1.4^{abcd}	-2.8 ± 3.8^{bc}	13.7 ± 0.7^{bcd}	28.6^{ghij}	182.1 ± 8.8^{de}	169.2 ± 8.2^{fg}	140.0 ± 6.8^{i}	163.8 ± 7.9^{ef}
L52.5_2	71.2 ± 1.9^{abc}	-3.3 ± 0.1^{abc}	15.0 ± 0.5^{fg}	28.9^{hij}	179.8 ± 3.0^{cd}	164.9 ± 2.9^{de}	124.7 ± 2.6^{cd}	156.5 ± 2.8^{d}
L60_1	72.9 ± 1.8^{def}	-3.3 ± 0.2^{abc}	15.0 ± 0.4^{fg}	27.1^{efg}	152.9 ± 4.5^{a}	139.5 ± 4.0^{a}	114.9 ± 3.3^{a}	135.8 ± 3.9^{a}
L60_2	73.3 ± 2.0^{efg}	-2.7 ± 3.2^{c}	13.2 ± 0.8^{bc}	26.7^{def}	184.2 ± 4.1^{efg}	171.6 ± 3.6^{ghi}	133.3 ± 3.0^{fg}	163.0 ± 3.5^{ef}
H20_1	73.5 ± 0.7^{efgh}	-3.0 ± 0.1^{abc}	15.8 ± 0.5^{hi}	26.5^{cdef}	186.7 ± 2.6^{fgh}	173.4 ± 2.3^{hij}	146.6 ± 1.7^{jk}	168.9 ± 2.2^{hij}
H20_2	73.9 ± 1.3^{fgh}	-3.6 ± 0.2^{abc}	13.4 ± 0.4^{bc}	26.1^{cde}	192.6 ± 1.6^{ijkl}	178.0 ± 1.7^{kl}	138.8 ± 1.9^{hi}	169.8 ± 1.7^{ij}
H25_1	73.6 ± 1.4^{efgh}	-3.1 ± 0.3^{abc}	16.2 ± 0.8^{ij}	26.5^{cdef}	188.9 ± 5.7^{hi}	175.0 ± 5.4^{ijk}	146.7 ± 4.5^{jk}	170.2 ± 5.2^{ijk}
H25_2	70.9 ± 1.2^{ab}	-3.9 ± 0.2^{ab}	13.6 ± 0.7^{bc}	29.2^{ij}	182.4 ± 6.8^{de}	175.9 ± 2.0^{jkl}	139.3 ± 2.3^{hi}	168.7 ± 2.4^{hij}
H30_1	71.0 ± 0.6^{ab}	-3.1 ± 0.3^{abc}	14.5 ± 0.5^{def}	29.0^{ij}	180.4 ± 4.^{1de}	166.7 ± 4.0^{ef}	139.7 ± 3.4^{i}	162.3 ± 3.8^{e}
H30_2	74.7 ± 1.1^{ghij}	-3.6 ± 0.3^{abc}	13.7 ± 0.6^{cde}	25.4^{abcd}	194.5 ± 2.8^{jkl}	182.2 ± 2.3^{m}	145.0 ± 1.8^{j}	173.9 ± 2.3^{kl}
H35_1	74.1 ± 0.9^{fghi}	-3.1 ± 0.2^{abc}	15.6 ± 0.7^{ghi}	25.9^{bcde}	190.8 ± 4.2^{hijk}	177.2 ± 3.7^{jkl}	149.0 ± 2.8^{kl}	172.3 ± 3.5^{jkl}
H35_2	76.0 ± 1.8^{j}	-3.8 ± 0.3^{abc}	13.4 ± 0.7^{bc}	24.1^{a}	190.2 ± 3.2^{hij}	175.6 ± 3.5^{jk}	133.9 ± 4.5^{g}	166.6 ± 3.5^{fghi}
H40_1	72.1 ± 1.5^{bcde}	-3.2 ± 0.3^{abc}	15.1 ± 1.1^{fgh}	27.9^{fghi}	182.8 ± 4.5^{def}	168.8 ± 4.3^{efg}	141.0 ± 3.7^{i}	164.2 ± 4.1^{efg}
H40_2	72.6 ± 2.1^{cdef}	-3.5 ± 0.4^{abc}	13.1 ± 0.8^{bc}	27.4^{efgh}	186.4 ± 2.8^{fgh}	173.5 ± 2.3^{hij}	135.6 ± 2.3^{gh}	165.2 ± 2.4^{efgh}
H45_1	70.1 ± 0.9^{a}	-3.6 ± 0.3^{abc}	11.5 ± 1.0^{a}	29.9^{j}	190.4 ± 4.3^{hijk}	175.6 ± 5.3^{ijk}	141.9 ± 3.5^{i}	168.2 ± 3.0^{hij}
H45_2	73.4 ± 1.1^{efg}	-3.6 ± 0.3^{abc}	14.6 ± 0.8^{ef}	26.6^{def}	184.5 ± 3.3^{efg}	170.6 ± 2.9^{fgh}	129.8 ± 2.5^{ef}	161.6 ± 2.8^{e}
H50_1	72.2 ± 1.0^{bcde}	-3.1 ± 0.5^{abc}	15.8 ± 1.3^{hi}	27.8^{fghi}	154.6 ± 3.1^{a}	139.8 ± 2.3^{a}	114.4 ± 1.6^{a}	136.3 ± 2.2^{a}
H50_2	72.2 ± 1.2^{bcde}	-3.3 ± 0.3^{abc}	14.9 ± 0.7^{fg}	27.8^{fghi}	175.8 ± 2.9^{c}	160.9 ± 2.3^{d}	121.7 ± 2.3^{bc}	152.8 ± 2.4^{d}

by different letters in each column are significantly different ($P < 0.05$)

Table 2 supplementary material: 5-hydroxymethylfurfural (HMF) and Maillard reaction products (MRP) content of bread samples (average value ± standard deviation for each replicated trial).

Sample code*	HMF (mg/kg dw)	Abs 420 nm (AU/g dw)	Abs 360 nm (AU/g dw)	Abs 280 nm (AU/g dw)
L30_1	4.15 ± 0.09^{a}	0.024 ± 0.001^{a}	0.097 ± 0.007^{a}	1.134 ± 0.065^{a}
L30_2	4.26 ± 0.28^{a}	0.025 ± 0.003^{a}	0.097 ± 0.006^{a}	1.182 ± 0.034^{a}
L37.5_1	14.95 ± 1.27^{ab}	0.055 ± 0.001^{abcd}	0.201 ± 0.007^{bc}	2.014 ± 0.100^{bc}
L37.5_2	6.45 ± 0.16^{a}	0.028 ± 0.002^{ab}	0.111 ± 0.009^{a}	1.277 ± 0.086^{a}
L45_1	16.86 ± 2.43^{abc}	0.055 ± 0.003^{abcd}	0.196 ± 0.016^{b}	1.981 ± 0.182^{bc}
L45_2	25.16 ± 2.24^{abcd}	0.068 ± 0.004^{bcd}	0.233 ± 0.008^{bcd}	2.188 ± 0.044^{c}
L52.5_1	55.72 ± 1.03^{d}	0.123 ± 0.020^{e}	0.382 ± 0.056^{e}	3.479 ± 0.492^{e}
L52.5_2	43.99 ± 1.83^{cd}	0.097 ± 0.010^{de}	0.311 ± 0.025^{de}	2.783 ± 0.256^{d}
L60_1	140.78 ± 4.61^{ef}	0.165 ± 0.499^{f}	0.018 ± 0.045^{f}	4.247 ± 0.339^{f}
L60_2	131.33 ± 0.72^{e}	0.169 ± 0.009^{f}	0.496 ± 0.011^{f}	4.003 ± 0.075^{f}
H20_1	4.42 ± 0.91^{a}	0.022 ± 0.002^{a}	0.090 ± 0.005^{a}	1.014 ± 0.030^{a}
H20_2	6.59 ± 0.18^{ab}	0.061 ± 0.005^{abcd}	0.152 ± 0.003^{ab}	1.200 ± 0.028^{a}
H25_1	24.52 ± 0.77^{abcd}	0.044 ± 0.003^{abc}	0.161 ± 0.009^{ab}	1.690 ± 0.075^{b}
H25_2	31.75 ± 1.99^{abcd}	0.074 ± 0.009^{cde}	0.229 ± 0.014^{bcd}	2.191 ± 0.057^{c}
H30_1	43.93 ± 2.63^{cd}	0.094 ± 0.008^{de}	0.285 ± 0.031^{cde}	2.641 ± 0.240^{d}
H30_2	35.25 ± 1.91^{bcd}	0.070 ± 0.001^{cd}	0.226 ± 0.008^{bcd}	2.218 ± 0.083^{c}
H35_1	151.24 ± 4.05^{ef}	0.207 ± 0.013^{g}	0.587 ± 0.021^{g}	4.663 ± 0.155^{g}
H35_2	166.12 ± 5.54^{f}	0.215 ± 0.008^{g}	0.584 ± 0.021^{g}	4.813 ± 0.323^{g}
H40_1	224.64 ± 13.36^{g}	0.256 ± 0.011^{h}	0.696 ± 0.027^{h}	5.470 ± 0.125^{h}
H40_2	211.99 ± 25.58^{g}	0.278 ± 0.009^{h}	0.746 ± 0.022^{h}	5.690 ± 0.169^{h}
H45_1	369.54 ± 42.55^{i}	0.462 ± 0.037^{i}	1.104 ± 0.029^{i}	7.850 ± 0.287^{i}
H45_2	315.37 ± 9.69^{h}	0.599 ± 0.033^{j}	1.429 ± 0.087^{j}	8.776 ± 0.411^{j}
H50_1	525.39 ± 65.73^{j}	0.753 ± 0.075^{l}	1.700 ± 0.124^{l}	9.749 ± 0.367^{k}
H50_2	333.12 ± 3.63^{h}	0.650 ± 0.046^{k}	1.523 ± 0.147^{k}	9.017 ± 0.714^{j}

* For sample identification see Table 1; replicated trials are identified by _1 and _2; values followed by different letters in each column are significantly different ($P < 0.05$)
